# The DNA methylation status of the serotonin metabolic pathway associated with reproductive inactivation induced by long-light exposure in Magang geese

**DOI:** 10.1186/s12864-023-09342-0

**Published:** 2023-06-26

**Authors:** Jiaxin Liu, Yanglong Xu, Yushuai Wang, Jinning Zhang, Yuting Fu, Sui Liufu, Danli Jiang, Jianqiu Pan, Hongjia Ouyang, Yunmao Huang, Yunbo Tian, Xu Shen

**Affiliations:** 1 College of Animal Science and Technology, Zhongkai University of Agriculture and Engineering, Guangzhou, 510225 China; 2 State Key Laboratory of Biocontrol, School of Life Sciences, Sun Yat-sen University, Guangzhou, Guangdong 510275 China

**Keywords:** DNA methylome, Gene expression, Long-light exposure, Reproductive inactivity, Magang geese

## Abstract

**Background:**

Domestic geese are seasonal breeders and have the lowest reproductive capacity among all poultry species. Magang geese is a topical short-day breeder, short photoperiod exposure stimulates its reproductive activity while long photoperiod inhibits. To explore epigenetic change that could influence reproductive activity, we performed whole genome bisulfite sequencing and transcriptome sequencing in the hypothalamus at three reproductive stages during long-light exposure in male Magang geese.

**Results:**

A total number of 10,602 differentially methylated regions (DMRs) were identified among three comparison groups. We observed that the vast majority of DMRs were enriched in intron regions. By integrating the BS-sequencing and RNA-seq data, the correlation between methylation changes of CG DMRs and expression changes of their associated genes was significant only for genes containing CG DMRs in their intron. A total of 278 DMR-associated DEGs were obtained among the three stages. KEGG analysis revealed that the DMR-associated DEGs were mainly involved in 11 pathways. Among them, the neuroactive ligand-receptor interaction pathway was significantly enriched in both two comparisons (RA vs.RD and RD vs.RI); the Wnt signaling pathway, apelin signaling pathway, melanogenesis, calcium signaling pathway, focal adhesion, and adherens junction were significantly enriched in the RA vs. RI comparison. In addition, the expression level of two serotonin-metabolic genes was significantly altered during reproductive axis inactivation by the methylation status of their promoter region (TPH2) and intron region (SLC18A2), respectively. These results were confirmed by Bisulfite sequencing PCR (BSP), pyrosequencing, and real-time qPCR, indicating that serotonin metabolic signaling may play a key role in decreasing the reproductive activity of Magang geese induced by long-light exposure. Furthermore, we performed a metabolomics approach to investigate the concentration of neurotransmitters among the three stages, and found that 5-HIAA, the last product of the serotonin metabolic pathway, was significantly decreased in the hypothalamus during RI.

**Conclusions:**

Our study reveals that the methylation status of the serotonin metabolic pathway in the hypothalamus is associated with reproductive inactivation, and provided new insight into the effect of DNA methylation on the reproductive regulation of the hypothalamus in Magang geese.

**Supplementary Information:**

The online version contains supplementary material available at 10.1186/s12864-023-09342-0.

## Introduction

Seasonal reproduction is an evolutionary strategy adopted by birds to maximize the survival of their offspring. Domestic geese are seasonal breeders and have the lowest reproductive capacity among all poultry species, causing significant economic losses in the poultry industry. Geese reproduction is day length-dependent and can be simply classified into short-day and long-day breeding [1]. Magang geese are typical short-day breeders, exposure to long days will lead to gonadal inactivity [2, 3]. Seasonal reproductive behavior in birds can be induced by day length, artificial photoperiod programs have been developed to induce out-of-season breeding in geese [2, 4, 5].

Birds perceive light by relying on the deep-brain photoreceptors (DBP) [6, 7]. Hypothalamus DBPs perceive and transmit light information and induce secretion of thyroid-stimulating hormone (TSH) from the pars tuberalis (PT), which reverses action on ependymal cells at the base of the third ventricle of the hypothalamus [7, 8]. Thyroid-stimulating hormone activates the type 2 deiodinase (dio2)/type 3 deiodinase (dio3) switching system and induces local Triiodothyronine (T3) release in the mediobasal hypothalamus [9]. Previous reports on long-day breeder quail and short-day breeder sheep showed that thyroid hormone synthesized from the medial basal hypothalamus (MBH) is the dominant signal controlling the activation of the reproductive axis [10, 11]. In chickens, the genetic mutation of the thyroid-stimulating hormone receptor gene (TSHR) contributes to the weakening of seasonal reproductive behavior [12, 13].

A major mechanism of seasonal reproduction is related to endocrine and molecular regulatory mechanisms on the hypothalamus-pituitary-gonadal axis [4, 5]. Hypothalamic thyroid hormone T3 stimulates the seasonal secretion of gonadotropin-releasing hormone (GnRH) which activates the reproductive system [13, 14]. A high level of T3 can induce functional recovery in dormant testis [9, 10].GnRH enters the pituitary gland through the portal vein and is transported to the pituitary, resulting in the release of follicle-stimulating hormone (FSH) and luteinizing hormone (LH) from the anterior pituitary gland, leading to gonadal maturation [14]. How photoperiod impacts on GnRH secretion remain controversial [15–17]. However, the different neuro-mediator systems such as neurotransmitters, vasoactive intestinal peptide (VIP) or thyroid hormone in hypothalamus have been shown to play a role in influencing complex endocrine processes related to photoperiodic regulation of seasonally reproductive activity [6, 18, 19].

Numerous studies have shown that photoperiodic adaptation produces phenotypes (such as seasonal reproduction) that are associated with changes in epigenetic markers [20–24].DNA Methylation is one of the important epigenetic mechanisms controlling behavior and physiology. Epigenetic modification participates in gene expression regulation that controls reproductive activities in seasonal reproductive breeds [24–26]. Epigenetic variation within brain tissues is an important factor to explain behavioral variation in avian [27]. Although transcriptome responses triggered by photoperiods have been extensively studied in seasonal reproductive breeds [28–30], little is known about photoperiod-induced transcriptome responses and epigenetic effects in short-day breeds. Whether epigenetic modifications mediate seasonal changes in gene expression in the geese hypothalamus are currently unknown but it is widely accepted that DNA methylation is inversely correlated with gene expression activation. An integrative analysis of both DNA methylation and gene expression is necessary for a full understanding of the involvement of epigenetics in seasonal reproduction in geese. In this study, we investigated epigenetic and transcriptional responses of Magang geese to long-light exposure using methylome and transcriptome profiling of the hypothalamus.

## Materials and methods

### Artificial photoperiodism and sample collection

Magang Geese is considered short-day breeder, and photoperiodism seriously affects its fertility. Magang Geese were obtained from the geese farm (Qingyuan, Guangdong). The artificial photoperiodism to induce the seasonal inactivation of Magang geese was shown (Supplementary Figures, Figure S1), reproductive activity stage (RA) indicated geese at their breeding seasons were maintained under a photoperiod of natural day length [(12 light (L):12 dark (D)], and then transferred to artificial long day condition (18 L: 6 D) for 17 days, long day promotes a decline of reproductive activity (RD) of the geese population, the laying rate in female geese at this stage is reduced to 17% compared to the laying period; two weeks later, the geese population were induced to reproductive inactivity stage (RI). Male geese used in the present study, three independent biological replicates were performed under each group (RA, RD and RI). Geese were euthanized by inhalation of carbon dioxide and cervical dislocation, which performed by laboratory technician who has extensive experience in application of techniques. Hypothalamus samples were collected and dissected.

### Bisulfite sequencing and bioinformatics analyses

Hypothalamus samples were collected and performed transcriptome sequencing and whole-genome bisulfite sequencing (BS-seq). Total RNA and Genomic DNA were immediatedly extracted from tissues. Bisulfite treatment were performed by using the Zymo EZ DNA Methylation-Gold™ Kit (ZYMO RESEARCH, CA, USA) with 500 ng genomic DNA, Bisulfite-treatmented DNA were purified to prepare whole-genome bisulfite sequencing libraries with the EpiGnome Kit (Epicenter, Madison, WI, USA). All libraries were sequenced at 100 bp paired-end reads on BGI MGISEQ-2000 platform (BGI, Shenzhen, Guangdong, China). Raw data were trimmed and filtered to remove adapters or low-quality base by using Trimmomatic v.0.36 [31]. Clean reads were mapped to the chromosome-level genome of the Lion-head goose (Anser cygnoides) (assembled by Zhongkai university of Agriculture and Engineering, data unpublished) using HISAT2 v2.1.0 software [32] with default parameters, and then unique mapping reads used for methylation analyses.

Bisulfite conversion efficiency were investigated to determine cytosines that were either methylated (false discovery rate, FDR % 0.05). Only cytosines covered by at least five sequencing reads were considered in the following test. DNA methylation level was defined by calculated the proportion of methylated cytosines among total cytosines relative to the total reads covering the sites was defined DNA methylation level. Sliding window analysis was performed to inspect the reproducibility between biological replicates (window size = 100 kb and step size = 50 kb) of methylation levels was conducted for each sample in all three sequence contexts. DMR detection was performed following the method of MethylKit [33]using a window size of 100 bp and a step size of 50 bp following the Benjamini–Hochberg multiple test correction [34]. Pearson’s correlation coefficient between methylation levels in pairs of biological replicates was estimated within three groups.

### RNA-seq and Differential gene expression analysis

Total RNA extracted as mentioned above was used for RNA-seq on Illumina HiSeq 4000 to generate 150 bp paired-end reads. Clean reads filtered by Fastp [35] were mapped to the geese genome using HISAT2 v2.1.0 software [32]with default parameter, DEseq2(v.1.26.0) [36] was performed to identification of DEGs, the threshold level was set with a standard. Differentially expressed genes (DEGs) were determined by DESeq2 (v.1.26.0) [36] requiring FDR < = 0.05, fold change > = 2. Each pairwise combination of the two reproductive stage was investigated. Principal component analysis (PCA) was conducted for each species using a regularized log2 transform of the normalized counts of all genes as generated by DESeq2 (v.1.26.0) [36]. Pearson correlation of gene expression (log2 of the normalized counts) between biological replicates was calculated using R v.3.6.2 (https://www.R-project.org). The DMR-related genes were performed Gene Ontology (GO) enrichment analysis by using Metascape (https://metascape.org/). KEGG pathway annotation was identified according to the KEGG database(https://www.genome.jp/kegg/) [37–39]. KEGG enrichment analysis was performed using KOBAS [40], and *p* < 0.05 was set as the threshold for significant enrichment. Primers of the differentially expressed genes used for qPCR validation are listed in Table S1.

### Target metabolomics for neurotransmitters in hypothalamus of geese

AB SCIEX QTRAP LC-MS/MS detection platform was used to detect the metabolites in the sample and quantify the content of the neurotransmitter in hypothalamic tissues among the three stages of geese during long light exposure, according to the proposal used in quail [18]. After whole hypothalamus homogenization, samples were processed for liquid Chromatograph Mass Spectrometer (LS-MS) detection within 24 h. Twenty-three neurotransmitter metabolites (shown in Table S2) were measured via Waters ACQUITY UPLC system (Waters, USA). Chromatography was performed on an ACQUITY UPLC BEH C18 column (2.1 × 100 mm, Waters, USA). Load 5 µL of each sample by the auto-sampler, the column temperature was 40 °C. Under mass spectrometry conditions, in a positive ion model using the following parameters: ion source temperature = 500◦C; ion spray voltage = 5,000 V; curtain gas (nitrogen) = 30 psi; both atomizing gas and auxiliary gas = 60 psi. Mass spectrometry was performed using the multiple reaction monitoring (MRM) scan method.

### Bisulfite sequencing PCR, pyrosequencing, and real-time PCR validation

We performed Bisulfite sequencing PCR (BSP) and pyrosequencing to validate the methylation change of two serotonin metabolism-related genes (*TPH2* and *SLC18A2*). Genomic DNA was extracted from hypothalamic tissues with MagPure Tissue DNA LQ Kit (D6321, Guangzhou Magen Biotechnology Co., Ltd.). The purified DNA was treated with bisulfite with Zymo EZ DNA Methylation-Gold™ Kit (ZYMO RESEARCH, CA, USA) according to the manufacturer’s protocol. Then the methylated DNA was amplified by BSP primer, PCR products was cloned into PGEM-T easy vector system (Promega, Madison, USA), and performed sanger sequencing, the methylation rate for each stage was calculated by the total number of 430 clones (117 clones in RA, 129 clones in RD, and 184 clones in RI). We performed pyrosequencing to validate the methylation level of the three genes (*TPH2*, *SLC18A2*, and *GPR26*). Primers were designed by PyroMark Assay Design 2.0 (https://www.qiagen.com/us/resources), and the 5’ end of the primer was biotinylated. Primers designed for bisulfite sequencing PCR, pyrosequencing and real-time PCR are listed in Table S1.

## Results

### DNA methylome profiles of the goose hypothalamus during HPG axis inactivation induced by long-light exposure

**Fig. 1:**
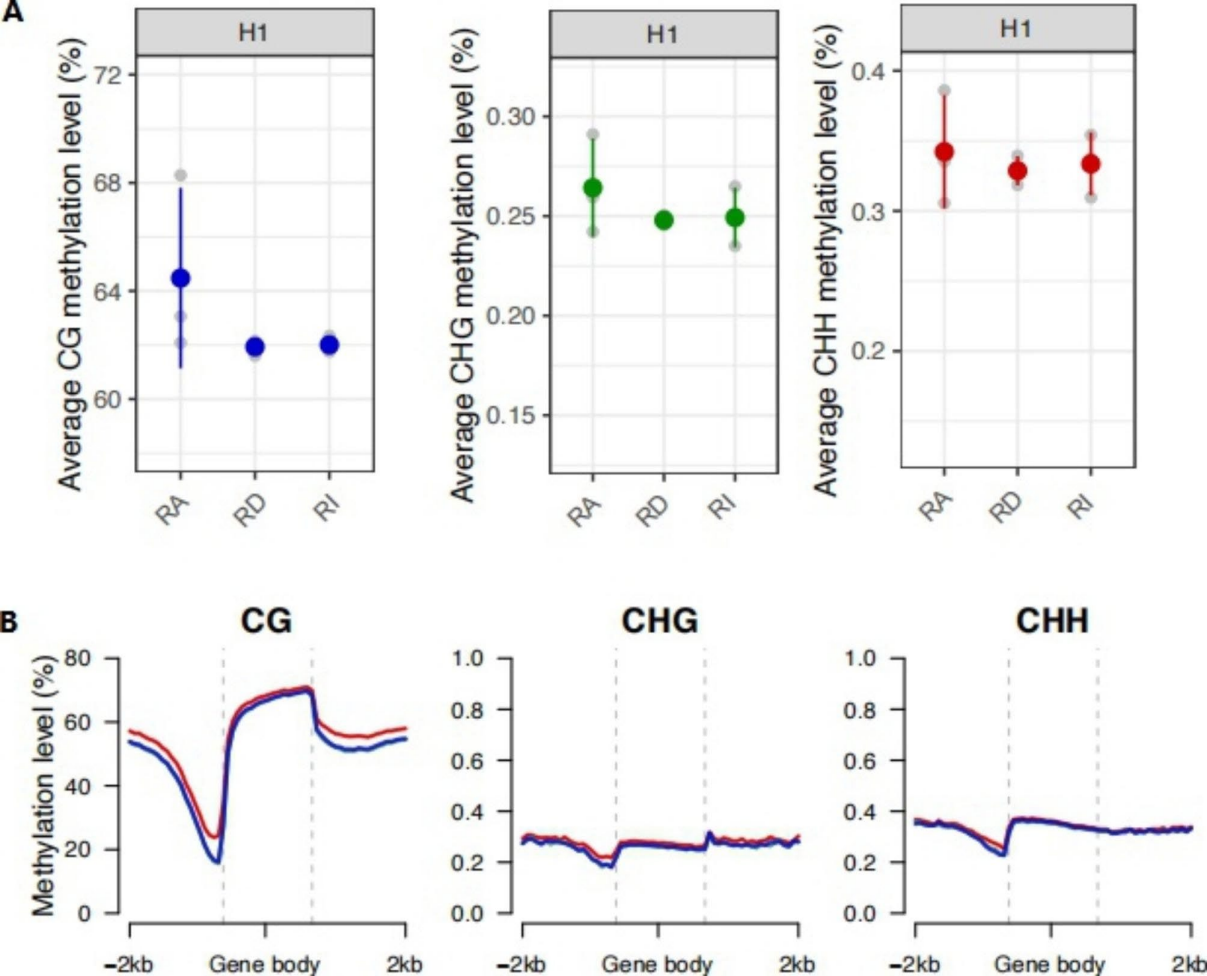
Global methylation profile of goose hypothalamus data. (A) The genome-wide methylation level of goose hypothalamus data. (B) Distribution of methylation levels across genes. The red curve, green curve and blue curve indicated the methylation level across genes in RA stage, RD stage, and RI stage respectively. Two tailed t-test: *, P < 0.05; **, P < 0.01

We determined methylation changes in the Magang goose genome underlying photoperiodism. Hypothalamus were collected for whole-genome bisulfite sequencing (BS-seq). Each stage had three biological replicates, with sequencing depths of ~ 30× (Table S3). BS-seq covered more than 85% of all cytosines. Methylation in the goose genome was found to exist in three types, including CG, CHG, and CHH (where H corresponds to A, T, or C). DNA methylation predominantly occurs in CG sites in the geese’s genome. CG-methylated cytosine sites accounted for the highest proportion in hypothalamus samples of geese, about 83% of all methylcytosines occurred in the CG context, while the proportion of methylated cytosine in the CHG and CHH context was ranging from 0.72 to 0.89% and 0.98 to 1.20%, respectively (Table 1). After long light exposure, the hypothalamus exhibited reduced levels of cytosine methylation in the CG context. The proportion of methylated cytosine in CG context varied greatly between replicates in RA (Table 1, F test, p < 0.05). No significant change in methylation levels in all three contexts was found among the three comparison groups (Fig. 1).

**Table 1:** Methylation levels in the goose genome in three sequence contexts (CG, CHG, and CHH, where H = A, T, or C)

Condition	Proportion of methylated sites				Genome-wide methylation level		
	CG	CHG	CHH		CG	CHG	CHH
RA	85.14 ± 2.14^a^	0.84 ± 0.05	1.10 ± 0.10		67.08 ± 2.97^a^	1.10 ± 0.06	1.22 ± 0.09
RD	83.76 ± 0.19^b^	0.80 ± 0.02	1.08 ± 0.04		64.71 ± 0.25^b^	1.08 ± 0.04	1.20 ± 0.04
RI	83.79 ± 0.10^b^	0.79 ± 0.08	1.07 ± 0.10		64.72 ± 0.31^b^	1.10 ± 0.03	1.23 ± 0.04

Note: The lowercase letters “a” “b” indicate the significant levels of variance in methylation levels across individuals between groups. A lowercase letter means *P* < 0.05, *F* test. The same letter means the difference is not significant, while different letters mean the difference is significant

**Fig. 2:**
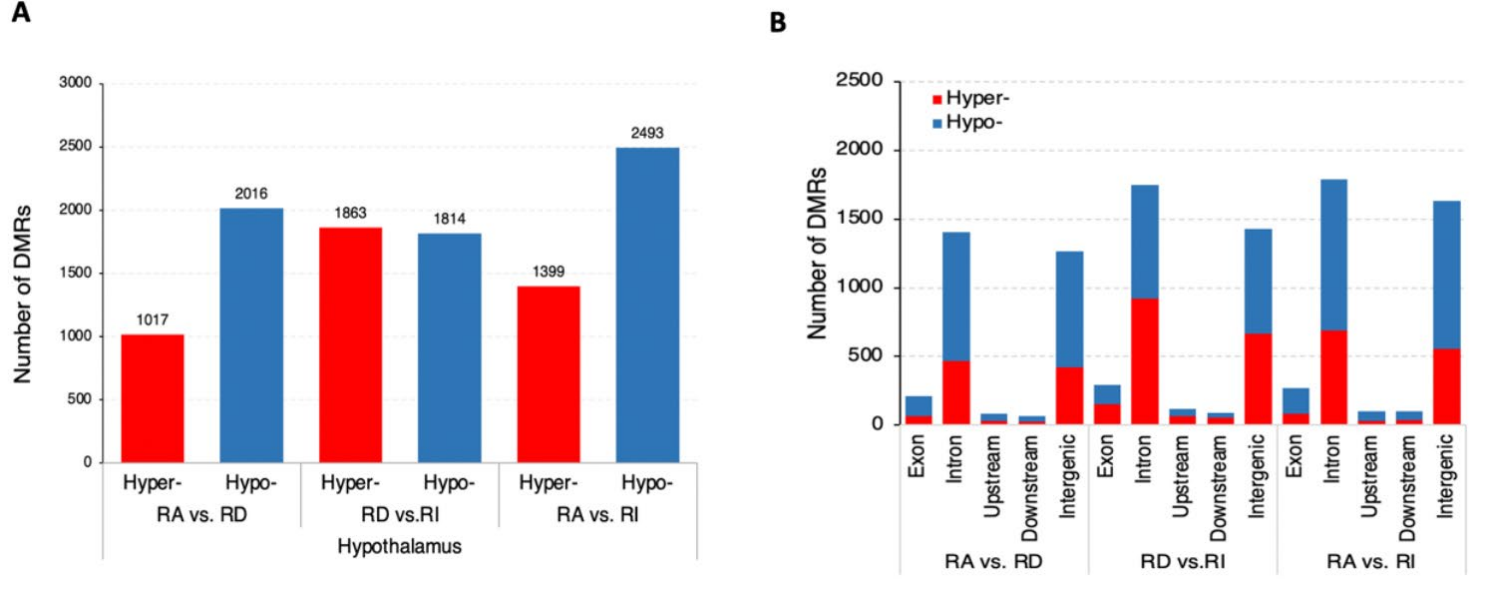
Genomic distribution of differentially methylated regions (DMRs) induced by long light exposure in the hypothalamus. (A) The number of differentially methylated regions (DMRs).(B) Distribution of DMRs in the goose genome. The numbers of hyper- (red) and hypomethylated CG, CHG, and CHH DMRs (blue) associated with genes (including exon, intron, upstream, downstream sequences, and intergenic)

Using a beta-binomial model [41], we identified 10,602 differentially methylated regions (DMRs) between any two-time points (RA vs. RD, RA vs. RI, and RD vs. RI) (Table S4) ; Among them, the total number of 3,033 DMRs (1017 hypermethylated and 2,016 hypomethylated) between RA and RD, 3,677 DMRs (1,863 hypermethylated and 1,814 hypomethylated) between RD and RI, and 3,892 (1,399 hypermethylated and 2,493 hypomethylated) between RA and RI (Fig. 2A) were identified. We then mapped the DMRs to genomic and genic features distribution of DMRs–the numbers of hypermethylated and hypomethylated CG, CHG, and CHH DMRs associated with genes were counted. We observed that the vast majority of DMRs were enriched within intron regions (Fig. 2B).

### Transcriptome change during HPG axis inactivation induced by long-light exposure

**Fig. 3:**
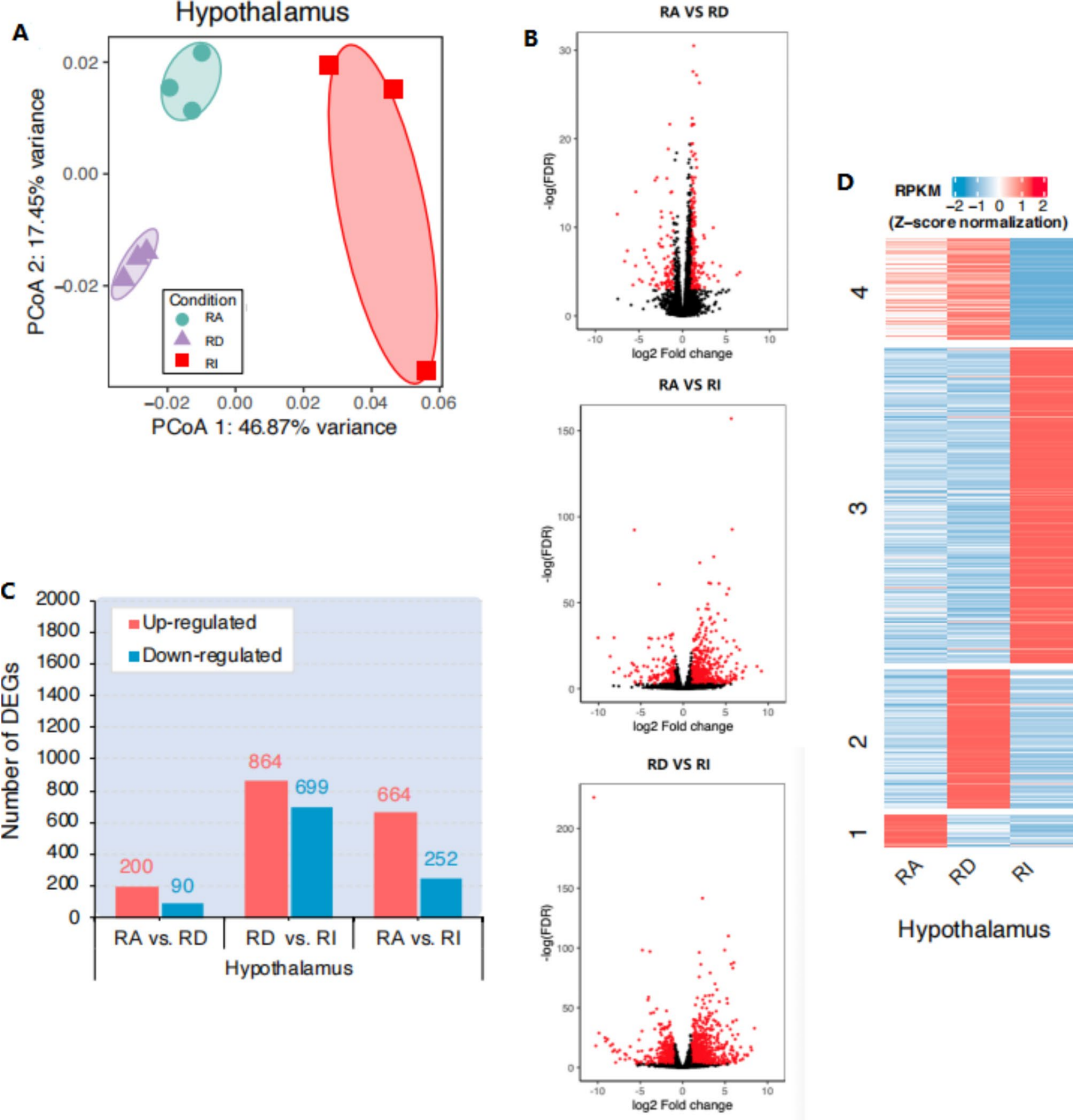
Transcriptome profile of goose hypothalamus. (A) Principal coordinate analysis (PCoA) of the goose hypothalamus data. (B) Volcano plot for the long photoperiod-induced changes of gene expression. Red points represent DEGs with at least a 2-fold change and an adjusted p-value (FDR) between pairs. (C) Differentially expressed genes (DEGs) identified in goose hypothalamus data. (D). Heat map of goose hypothalamus data

**Fig. 4:**
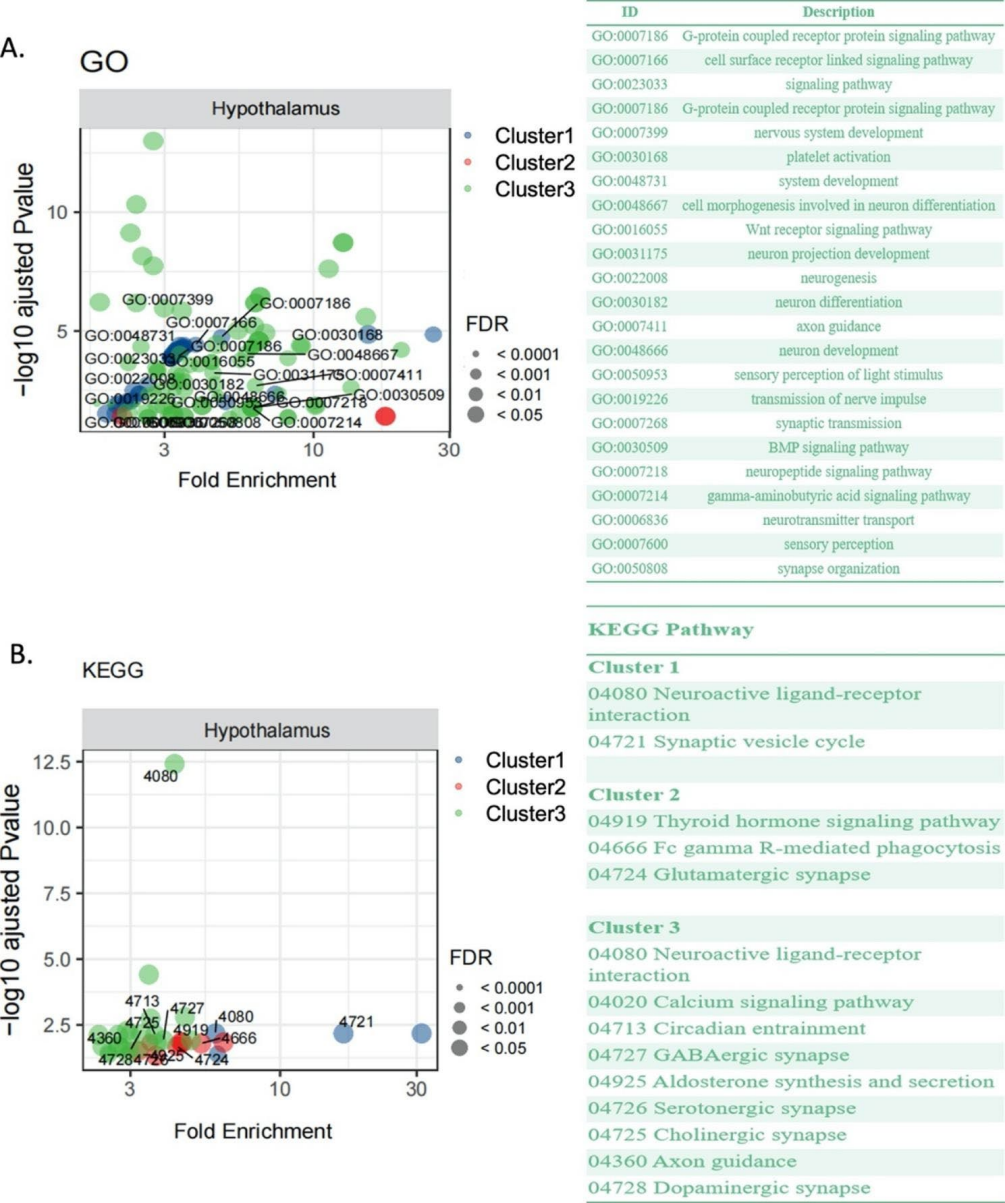
The GO terms (A) and Kyoto Encyclopedia of Genes and Genomes (KEGG) pathway enrichments (B) of the DEGs. (A) GO terms enriched in the differentially expressed genes among the cluster 1, 2 and 3. (B) KEGG enriched in the differentially expressed genes among cluster 1, 2 and 3

To assess the potential impact of methylation changes on gene expression, RNA-seq was conducted to identify the differentially expressed genes (DEGs) (Table S5 and Table S6). Principal component analysis (PCA) of the normalized count data separated the three stages (Fig. 3A). We identified 290 DEGs between RA and RD, 1,563 DEGs between RD and RI, and 916 DEGs between RA and RI (Fig. 3B, C). Cluster analysis of DEGs identified four statistically significant clusters (Fig. 3D);

Gene Ontology (GO) analysis revealed that the DEGs in cluster1 significantly enriched in G-protein coupled receptor protein signaling pathway; In cluster 2, DEGs were associated with transmembrane transport and regulation of cell activation; GO categories represented in cluster 3 were associated with the cell surface receptor linked signaling pathway, G-protein coupled receptor protein signaling pathway, and neuron system development (Fig. 4A).

To further understand the role of DEGs in the functional regulation of the hypothalamus during seasonal inactivation, signaling pathways were analyzed by the Kyoto Encyclopedia of Genes and Genomes (KEGG). The results showed that the highlighted pathways significantly overrepresented in cluster 1 were neuroactive ligand-receptor interaction (VIPR1, TRHR, P2RX3, PTGDR, CCKAR) and synaptic vesicle cycle (TPH2, SLC6A4, SLC18A2, DDC). The highlighted pathways significantly overrepresented in cluster 2 were thyroid hormone signaling pathway (DIO2, SRC, PFKFB2, FOXO1, HIF1A, ITGAV, NCOA3, PRKCB, SLC9A1), and glutamatergic synapse. The highlighted pathways significantly overrepresented in cluster 3 were neuroactive ligand-receptor interaction, calcium signaling pathway, and ECM-receptor interaction (Fig. 4B). The synaptic transmission pathway was significantly changed in cluster 3, enriched in several synaptic types, including GABAergic synapse, serotonergic synapse, dopaminergic synapse, and cholinergic synapse. For validation, six genes related to hypothalamic neurotransmitters metabolic were selected to be analyzed by qRT-PCR (Supplementary Figures, Figure S2).

### DMR-associated DEGs during HPG axis inactivation induced by long-light exposure

**Fig. 5:**
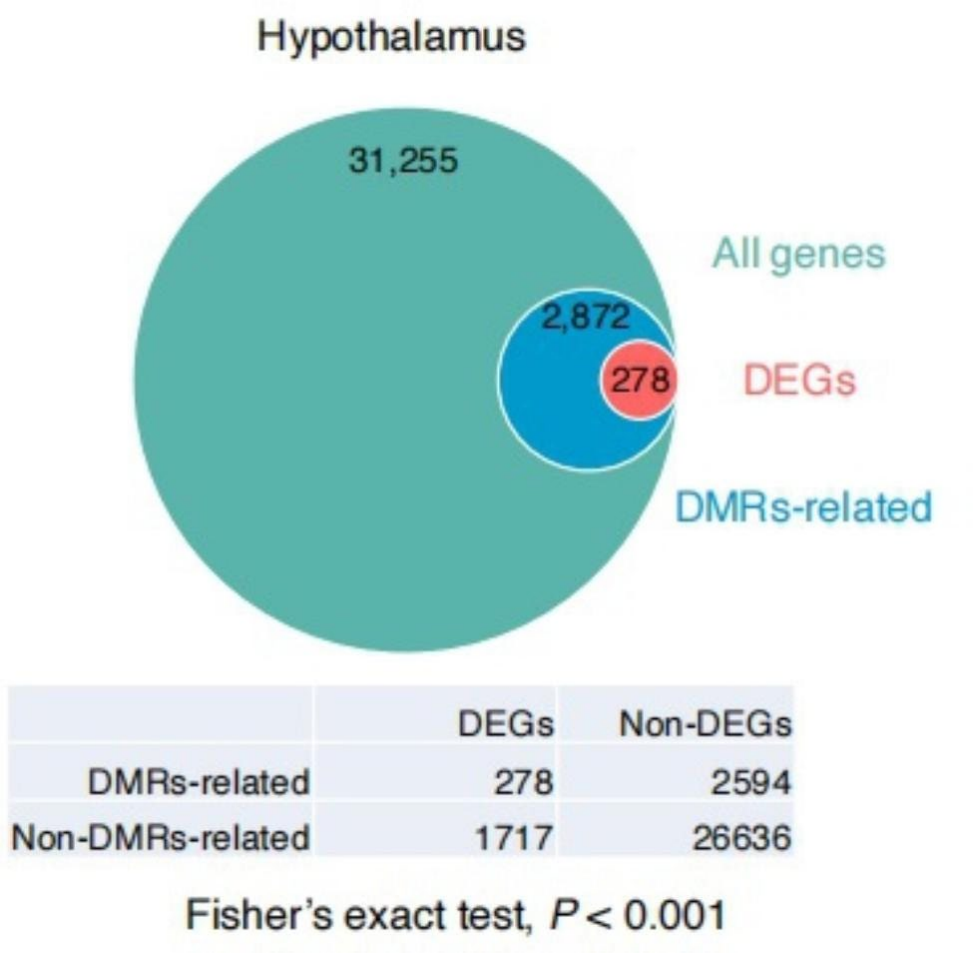
DMR-related genes for DEGs in the goose hypothalamus. Venn diagrams showed the DMRs-related DEGs identified in the hypothalamus. Fisher’s exact test showed that DMRs-related genes were more likely to be differentially expressed (*P* < 0.001)

To evaluate the potential impact of methylation changes on gene expression during the HPG axis inactivation, we examined the distribution of the DMR-associated DEGs (Fig. 5). A total number of 278 DMRs-associated DEGs were identified among three stages in the hypothalamus, Fisher’s exact test showed that DMRs-associated genes were more likely to be differentially expressed (*P* < 0.001), indicating the regulatory effect of DNA methylation on gene expression. Among them, thirty-three DMR-associated DEGs were identified in the RA vs. RD comparison (82.6% of DMRs located in the intron, 6.5% of DMRs located in the upstream of the gene, 4.3% located in the downstream of gene, and 6.5% located in the exon); One hundred and thirty-seven DMR-related DEGs were identified in the RD vs. RI comparison (75.3% of DMRs located in the intron, 8.4% located in the upstream of the gene, 4.0% located in the downstream of gene, and 12.3% located in exon); In the RA vs. RI comparison, 108 DMR-associated DEGs were identified (77.3% of DMRs located in the intron, 2.8% located in the upstream of the gene 1.7% located in the downstream of gene, and 18.2% located in the exon) (Table S7).

**Fig. 6:**
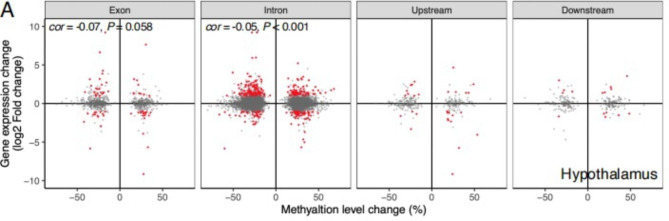
Influences of DMRs on the expression of genes. Quadrant plot of methylation changes of differentially methylated regions (DMRs) and expression changes of the DMR-associated differentially expressed genes during HPG inactivation. The x-axis represents the methylation difference of DMRs among comparison groups. The y-axis indicates the expression difference (log2-fold change) of the DMR-associated genes among the three comparison groups. The red points indicate genes with significant (Benjamini-Hochberg FDR < 0.05) differential expression. Cor indicates Pearson’s correlation coefficient. Only significant (p < 0.05) correlation coefficients are shown

To evaluate the potential impact of methylation changes on gene expression in the hypothalamus during HPG axis inactivation in geese, we examined the expression of genes that are associated with DMRs in the hypothalamus. The quadrant plot showed the correlation between the methylation changes of differentially methylated regions (DMRs) and expression changes of the DMR- associated differentially expressed genes during HPG axis inactivation (Fig. 6). The results showed that the genes containing DMRs in their introns have a weak negative correlation between the methylation changes and the expression changes (Pearson’s correlation, *r* = -0.05, P < 0.0001) (Fig. 6). No significant negative correlation was detected between the gene containing DMRs in their exon and expression changes of their associated genes (Pearson’s correlation, *r* = -0.07, P = 0.058). Therefore, methylation changes during HPG axis inactivation have a weak negligible impact on genome-wide gene expression in the hypothalamus of geese.

To further investigate the biological function in which DMR-associated DEGs might be involved, we performed a KEGG pathway analysis (Table 2). KEGG analysis revealed that the DMR-associated DEGs were mainly involved in 11 pathways. Among them, the neuroactive ligand-receptor interaction pathway was significantly enriched in both two comparisons (RA vs.RD and RD vs.RI); the Wnt signaling pathway, apelin signaling pathway, melanogenesis, calcium signaling pathway, focal adhesion, and adherens junction were significantly enriched in the RA vs. RI comparison (Table 2).

**Table 2:** The KEGG pathway enrichment of DMR-related genes

Compared Groups	Term	P-Value	Enriched Genes
**RA Vs RD**			
	Neuroactive ligand-receptor interaction	9.80E-02	*Adrb2*, *OPRD1*, *VipR1*
**RD Vs RI**			
	Calcium signaling pathway	5.80E-05	*CACNA1G*, *CAMK1G*, *Camk2a*, *PDGF*, *Ppp3cb*, *PTK2B*, *RET*, *NTRK1*
	MAPK signaling pathway	3.10E-02	*CACNA1G*, *DUSP5*, *NTRK1*, *PDGFC*, *Ppp3cb*
	Wnt signaling pathway	3.30E-02	*AXIN1*, *Camk2a*, *PRICKLE1*, *Ppp3cb*
	Neuroactive ligand-receptor interaction	7.60E-02	*EDN3*, *Gabrr1*, *GRIN2B*, *GRM4*, *Lpar3*
**RA Vs RI**			
	Wnt signaling pathway	9.50E-06	*SMAD3*, *SMAD4*, *Wnt3*, *Wnt8b*, *AXIN1*, *Camk2d*, *Camk2g*, *PRICKLE1*, *TCF7L2*
	Apelin signaling pathway	2.00E-03	*GNG2*, *PPARGC1A*, *SMAD3*, *SMAD4*, *ITPR1*, *PLAT*
	Melanogenesis	4.40E-03	*Wnt3*, *Wnt8b*, *Camk2d*, *Camk2g*, *TCF7L2*
	Calcium signaling pathway	2.30E-02	*CACNA1E*, *Camk2d*, *Camk2g*, *ITPR1*, *LHCGR*, *PDGFC*
	Focal adhesion	5.10E-02	*PAPGEF1*, *Dock1*, *ITGA8*, *ITGAV*, *PDGFC*
	Adherens junction	9.50E-02	*SMAD3*, *SMAD4*, *TCF7L2*

### DMR-associated DEGs are involved in neurotransmitter metabolism during HPG axis inactivation induced by long-light exposure

We investigated the biological function of the top 10 DMR-associated DEGs in the comparison of RA and RI, and found that two genes (*TPH2* and *SLC18A2*) were involved in neurotransmitter metabolism. Compared with RA, DNA methylation levels of the TPH2 gene and SLC18A2 gene in RI increased by 31.85% and 31.99%, respectively. Significantly elevated DNA methylation levels reduced the gene expression level of *TPH2* and *SLC18A2* in the RI. BSP and Pyrosequencing was performed to validate the high methylation level of the TPH2 and SLC18A2 gene in the RI stage, and measure the methylation level change of the GPR26 gene between RA and RD stage (Table 3, Table S8, and Supplementary Figures, Figure S3). In addition, we performed targeted quantification of neurotransmitters in the goose hypothalamus among three stages, and found the concentration of 5-hydroxyindole acetic acid (5-HIAA), which was the last product of 5-HT metabolism significantly declined in the RI stage compared to the RA stage (Table 4). Moreover, Chronic exposure to different photoperiods could alter the number of neurotransmitters in the hypothalamus, we observed that the concentration of histidine was significantly increased in RD, but showed no significant difference between RA and RI.

**Table 3:** Methylation level of TPH2, SLC18A2, and GPR26 validated by pyrosequencing

			Pos. 1	Pos. 2	Pos. 3	
Assay	Condition	Sample ID	Meth. (%)	Meth. (%)	Meth. (%)	Mean
	RA	RA1	65.1	85.52	43.39	64.67
		RA2	62.19	82.84	41.32	62.12
TPH2		RA3	53.09	68.16	35.5	52.25
	RI	RI1	100	95.12	78.14	91.09
		RI2	98.15	82.04	65.16	81.78
		RI3	100	94.15	78.04	90.73
	T-test	Ra vs RI				0.001
	RA	RA1	48.14	37.12	/	26.40
		RA2	43.35	43.42	/	43.39
		RA3	41.65	31.57	/	36.61
SLC18A2	RI	RI1	52.66	42.42	/	47.54
		RI2	58.27	58.69	/	58.48
		RI3	52.58	40.78	/	46.68
	T-test	Ra vs RI				0.034
	RA	RA1	64.38	66.34	/	65.36
		RA2	58.89	58.62	/	58.75
GPR26		RA3	55.78	56.75	/	56.27
	RD	RD1	7.62	21.99	/	14.81
		RD2	7.25	20.33	/	13.79
		RD3	8.61	20.95	/	14.78
	T-test	RA vs RD				3.76E-05

Notes: one-tailed T-test was performed to compare the mean of methylation level between two groups

**Table 4:** Concentration of neurotransmitters during HPG axis inactivation in the goose hypothalamus (n = 3)

Stage	GABA	Gln	Glu	His	NE	Tyr	5-HIAA	L-Tryptophan
RA	2,415.60 ± 369.19	1,449.20 ± 120.06	1,570.04 ± 363.20	152.53 ± 37.70^ab^	3.26 ± 0.77	294.33± 45.51	0.84 ± 0.23^a^	53.51 ± 10.18
RD	2,512.09 ± 294.27	1,255.54 ± 318.02	1,486.93 ± 161.37	196.20± 13.68^a^	2.95 ± 0.63	322.29± 41.36	0.70 ± 0.33^ab^	60.00 ± 4.70
RI	2,531.18 ± 353.86	1,361.47 ± 463.25	1,521.85 ± 130.14	162.04± 18.52^b^	3.32 ± 0.52	298.07± 45.08	0.32 ± 0.09^b^	55.08 ± 6.80

Note: GABA indicated 4-Aminobutyric acid; Gln indicated L-Glutamine; Glu indicated L-Glutamic acid; His indicated L-histidine; NE indicated Noradrenaline hydrochloride; Tyr indicated L-Tyrosine; 5-HIAA indicated 5-Hydroxyindole-3-acetic acid; Trp indicated L-Tryptophan.

The lowercase letters “a” “b” indicate the significant levels of variance in methylation levels across individuals between groups. A lowercase letter means P < 0.05, F test. The same letter means the difference is insignificant, while different letters mean the difference is significant

## Discussion

The hypothalamus is an integral part of the central nervous system that integrates environmental stimuli and endogenous signals [21]. Recent findings indicate that epigenetic modifications are essential in regulating and timing seasonal rhythms [42, 43]. DNA methylation was important in regulating the seasonal timing of reproduction in Great Tits [44]. Short photoperiods inhibit reproductive activity in hamsters because hypermethylation altered the mRNA expression level of a critical gene involved in the thyroid hormone synthesis pathway in the hypothalamus [24]. In songbirds, the hypothalamus showed epigenetic differences between the non-migrant and migrant periods, which were associated with photoperiodic changes [45]. Long light exposure induces reproductive inactivation in Magang geese; however, the epigenetic changes concerned with seasonal reproduction in the hypothalamus are still unknown.

At the epigenetic level, DNA methylation predominantly occurs in CG sites in the geese genome. Prolonged light exposure did not change methylation levels in all three contexts in Magang geese. In contrast, long light induced the expression level of DNA methyltransferase 3α (DNMT3A) in the hypothalamus was significantly increased in the hamster, a long-day breeder [46]. The most interesting finding was that gene containing DMRs in their introns was observed to have a weak negative correlation between the methylation changes and the expression changes in the hypothalamus. These results indicate that intron methylation may have important regulatory functions in the hypothalamus that are associated with reproductive inactivation during long light exposure. Recently, the regulatory role of non-coding variants at the intron region of TSHR contributes to the regulation of seasonal reproduction in Atlantic herring [47].

At the expression level, Photoperiodism impacted gene expression levels in the hypothalamus [4, 5, 48]. Gene expression in the hypothalamus was associated with increased TSHβ and dio2 mRNA levels and decreased dio3 mRNA levels under long light conditions [48]. As expected, the expression level of dio2 observed a significant increase in RD after long light exposure in the hypothalamus of Magang geese. By comparing the transcriptome differences between RA and RI, we found that the effects of prolonged light stimulation were mainly related to the serotonin (5-HT) /dopamine (DA)- Vasoactive intestinal polypeptide (VIP) system. In avian, the reproductive behavior was strictly controlled by the hypothalamic-pituitary-gonadal axis, and the hypothalamic neurotransmitters 5-HT, DA, and VIP have important effects on the secretion of pituitary prolactin [49–53].

Compared to RA, two serotonin metabolism genes (SLC18A2 and TPH2) had the largest change in expression level in RI. As the rate-limiting enzyme of 5-HT synthesis, TPH2 was widely expressed in the brain and controlled serotonin synthesis. The mRNA expression pattern in TPH2 is affected by the photoperiod [54, 55]. SLC18A2 is a key regulator of the monoamine neurotransmitter systems, which can package serotonin and dopamine neurotransmitters in the cytoplasm into vesicles for release [56]. Zebrafish individuals with homozygous mutants showed abnormal responses to light changes and affected brain development [57]. We also observed that the mRNA expression level of GnRH-1 significantly increased in the RI stage, suggesting that long light exposure inhibited the GnRH signaling is inhibited in short-day breeders [58]. Long days stimulated the mRNA expression level of GnRH-1 [24], in long-day breeder starlings, GNRH-1 mRNA-expressing cells were significantly greater in breeding birds than in nonbreeding birds [59].

Long light exposure associated with reproductive inactivation in Magang geese is accompanied by alterations in the epigenome. DMR-associated DEGs in the hypothalamus, and the methylation level change in the intron had a significant negative correlation with gene expression. Compared to the RA stage, VIPR1 showed the most decreased expression levels and hypomethylated DMRs responded to long light exposure in the RD stage. The VIPR1 gene is reported to be significantly associated with chicken reproduction [60–62]. VIP is known to play a vital role in internal SCN timekeeping, and VIP was the first activated following a long light stimulation [63, 64]. GPR26 showed significantly decreased expression levels, and the most hypermethylated DMRs responded to long light exposure. GPR26 is a central brain-specific orphan GPCR whose endogenous ligand remains unclear. Genetic deletion of GPR26 leads to anxiety and depression-like behaviors [65]. Compared to RA, TPH2, and SLC18A2 showed significantly decreased expression levels and the most hypermethylated DMRs responded to long light exposure in the RI stage. Both genes were involved in serotonin metabolism, we validated the CpG site in both two genes and showed the hypermethylation level occurred upstream of the TPH2 gene and in the intron of SLC18A2. To directly assess whether these alterations have any functional consequence on serotonin metabolism, neurotransmitter data showed the concentration of 5-HIAA decreased in the RI stage in the hypothalamus. In male rhesus macaques, the concentration of CSF 5-HIAA significantly increases in the breeding season [66, 67]. In humans, 5-HT turnover shows circannual variation by season [68].

Taken together, our results reveal a functional association between serotonin metabolism in hypothalamus and reproductive inactivation in Magang geese during long light exposure.

## Electronic supplementary material

Below is the link to the electronic supplementary material.

Supplementary Material 1

Supplementary Material 2

Supplementary Material 3

Supplementary Material 4

Supplementary Material 5

Supplementary Material 6

Supplementary Material 7

Supplementary Material 8

Supplementary Material 9

## Data Availability

The data sets supporting the results of this article were included within the article and additional files. The raw sequence data reported in this paper have been deposited in the Genome Sequence Archive (Genomics, Proteomics & Bioinformatics 2021) at National Genomics Data Center (Nucleic Acids Res 2022), China National Center for Bioinformation / Beijing Institute of Genomics, Chinese Academy of Sciences (Bio-Project of Bisulfite sequencing data and RNA-seq data: PRJCA013343; GSA accession of Bisulfite sequencing data and RNA-seq data: CRA008993) that are publicly accessible at https://ngdc.cncb.ac.cn/gsa.
