# Supplementary material for: The DNA methylation status of the serotonin metabolic pathway associated with reproductive inactivation induced by long-light exposure in Magang geese
Source: BMC Genomics. 2023 Jun 26;24:355. doi: 10.1186/s12864-023-09342-0 (PMC10294383; doi:10.1186/s12864-023-09342-0)
Supplement: Supplementary file 1 — Supplementary Material 1 [file 12864_2023_9342_MOESM1_ESM.pdf]

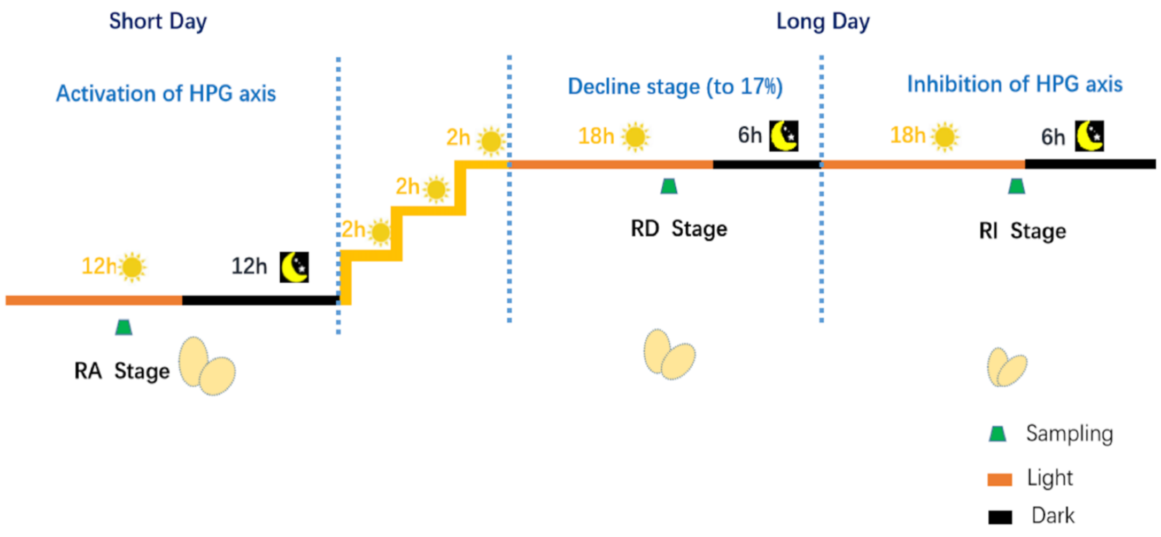

Figure S1 The artificial photoperiodism in exprement design. Geese at their breeding seasons (**RA stage**) were maintained under a short-day photoperiod of natural day length [(12 light (L):12 dark (D)]; After sampling, geese were transferred to implemented artificial lighting conditions, beginning by increasing light exposure by 2 hours each day until reached the maximum of 18 hours of light per day. Geese were maintained under artificial long-day conditions (18 L: 6 D) for 17 days; the artificial long-day promotes a decline of reproductive activity (**RD stage**) of the geese population when the laying rate in female geese at this stage is reduced to 17% compared to the laying period, we sampled the hypothalamus tissues; Two weeks later, the geese population was induced to the reproductive inactivity stage (**RI stage**). The green trapezoid represents the time the sample was collected; the orange rectangles represent the hours of light exposure, while the black rectangle represents the hour of darkness.

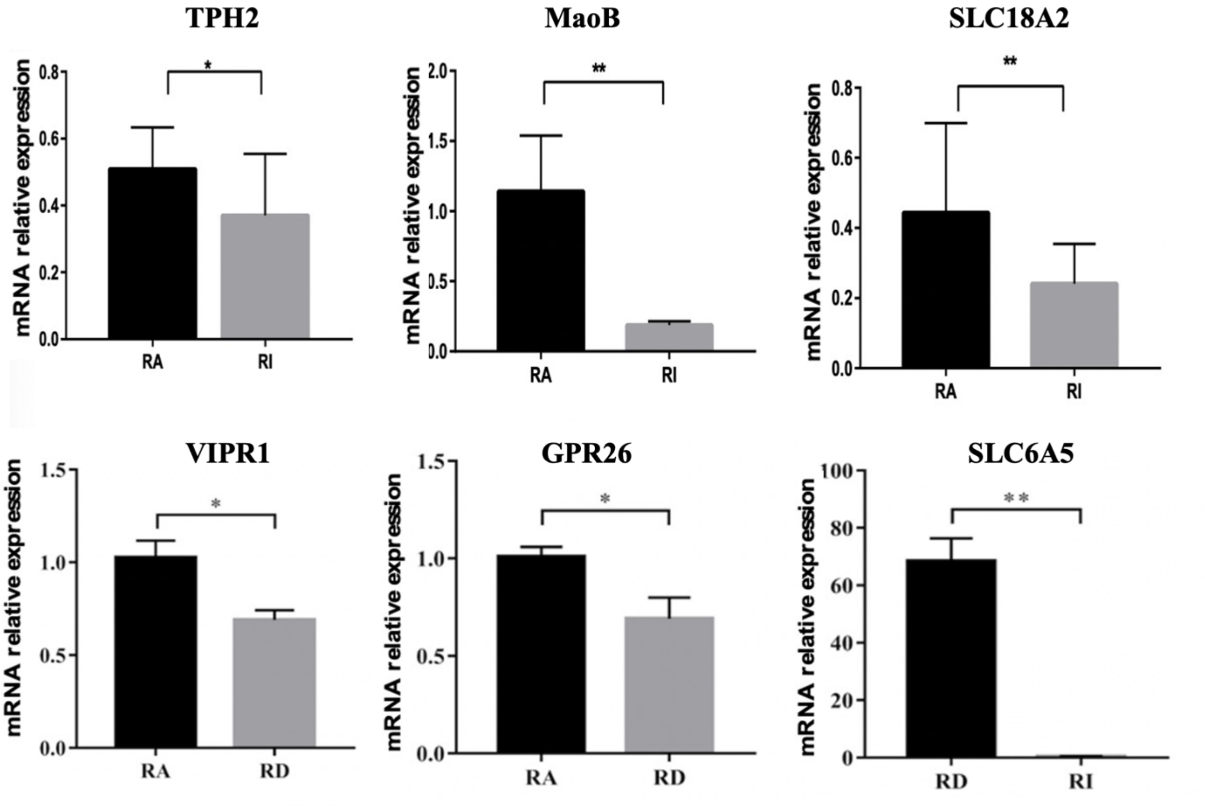

Figure S2 Real-time PCR validation for gene expression difference between the two comparison groups in the hypothalamus. Two-tailed T-test was performed to compare the mean expression level between the two groups; P values less than 0.05 are given one asterisk; while P values less than 0.01 are given two asterisks.

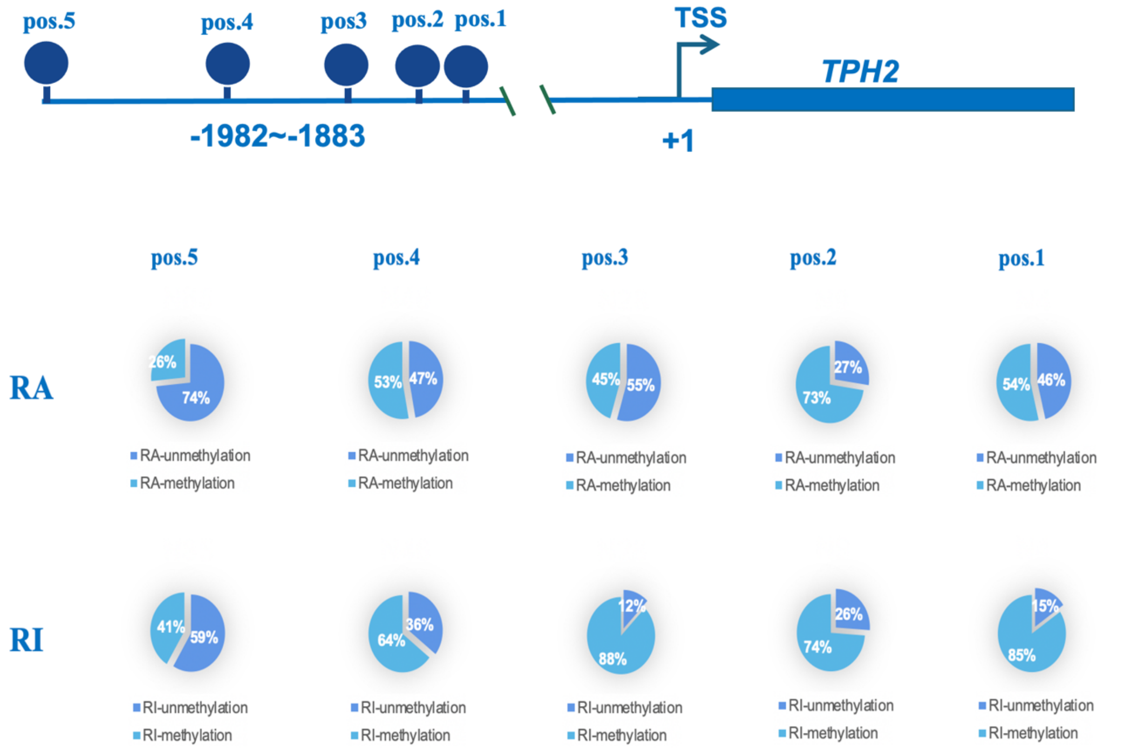

Figure S3 Bisulfite sequencing PCR validated the methylation level of TPH2. By bisulfite conversion, all unmethylated cytosine (C) is converted to uracil (U) while methylated cytosine remains unchanged in the DNA sequence. We validate 5 methylation sites among RA(number of individuals =3) and RI(number of individuals =3). PCR products were cloned into a TA-cloning vector (PMD19-T). 301 Clones were sequenced by sanger sequencing (117 clones in RA, and 184 clones in RI). We performed methylation calling and visualization of the results. Since BS-seq changes unmethylated cytosines (C) to thymines (T), subsequent analysis steps focus on counting the number of C to T conversions and quantifying the methylation proportion per base. "1" means the C-to-T conversions, and "0" means the unmethylated cytosines (C). We Calculated the average methylation rate between RA and RI groups.
