# Supplementary material for: The DNA methylation status of the serotonin metabolic pathway associated with reproductive inactivation induced by long-light exposure in Magang geese
Source: BMC Genomics. 2023 Jun 26;24:355. doi: 10.1186/s12864-023-09342-0 (PMC10294383; doi:10.1186/s12864-023-09342-0)
Supplement: Supplementary file 2 — Supplementary Material 2 [file 12864_2023_9342_MOESM2_ESM.docx]

Table S1 Pimers used for the real-time quantitative PCR, Bisulfite sequencing PCR and Pyrosequencing.

| Experiments | Genes | Primer sequence (5’-3’)^1^ | Length (bp) |
| --- | --- | --- | --- |
|  | *ACTB* | F: 5'-CCTCTTCCAGCCATCTTTCTT-3' | 110 |
|  |  | R: 5'-TGTTGGCATACAGGTCCTTAC-3' |  |
|  | *TPH2* | F: 5'-CAGTGAGACCAGTTGCTGGA-3' | 156 |
|  |  | R: 5'-GAGGCACATGTCCCAAGAGT-3' |  |
| RT-PCR | *SLC18A2* | F: 5'-CCATCTGATTGCCCTAAGGA-3' | 238 |
|  |  | R: 5'-CTTGGAGGGACCTTGCAATA-3' |  |
|  | *MaoB* | F: 5'-AGTGGAGCGGATACATGGAG-3' | 188 |
|  |  | R: 5'-TAGCAGTCCTGGCACAGATG-3' |  |
|  | *VIPR1* | F: 5'-CTGGTGGAAGGGCTGTATCT-3' | 160 |
|  |  | R: 5'-CCCAGCACCCAACATTGAAA-3' |  |
|  | *GPR26* | F: 5'-TCCTGCACCCTGTACAACAA-3' | 183 |
|  |  | R: 5'-CACCAGGGTCTGCATAGTGA-3' |  |
|  | *SLC6A5* | F: 5'-CCCGTACCTGGCCTTTAAGA-3' | 197 |
|  |  | R: 5'-TCAGGACGGAGATGATGAGC-3' |  |
| Pyrosequencing^2^ | *SLC18A2* | F: 5'-GAGTTTTAAATAAGTATGGAGTTATTGG-3' | 216 |
|  |  | R: 5'-Biotin-AAATCCTTCAACTCCAACAATTA-3' |  |
|  |  | S: 5'-AGTATGGAGTTATTGGT-3' |  |
|  | *TPH2* | F: 5'-GTTTGTTATGTTTGAGATATTTGGATATAG-3' | 117 |
|  |  | R: 5'-Biotin-AAACCTAAAATAAAACTTATTTTTATCACT-3' |  |
|  |  | S: 5'-GTTTGAGATATTTGGATATAGT-3' |  |
|  | *GPR26* | F: 5'-TTGTTTGATTGGGGTTATAGGTATG-3' |  |
|  |  | R: 5'-Biotin-AAAACAACTAATCACCAAATTTCACT-3' |  |
|  |  | S: 5'-GGGTTATAGGTATGGTTTT-3' |  |
| Bisulfite sequencing PCR | *TPH2* | F: 5'-AAATAGAGTTGTGATAGATAGTAAGGT-3' | 345 |
|  |  | R: 5'-AACCTTATATCCAAAATTACCAAAAA-3' |  |

Notes: ^1^ F: indicated forward primers; R: indicated reverse primers; S: indicated sequencing primers.

^2^ The 5' end of the pyrosequencing primers were biotinylated
