# Supplementary material for: The DNA methylation status of the serotonin metabolic pathway associated with reproductive inactivation induced by long-light exposure in Magang geese
Source: BMC Genomics. 2023 Jun 26;24:355. doi: 10.1186/s12864-023-09342-0 (PMC10294383; doi:10.1186/s12864-023-09342-0)
Supplement: Supplementary file 3 — Supplementary Material 3 [file 12864_2023_9342_MOESM3_ESM.docx]

Table S2 List of neurontransmitts detected in hypothalamus

| No | metabolite | abbreviation |
| --- | --- | --- |
| 1 | 4-Aminobutyric acid | GABA |
| 2 | Histamine | HisA |
| 3 | Picolinic acid | PA |
| 4 | Tyramine | TyrA |
| 5 | Acetylcholine chloride | Ach |
| 6 | L-Glutamine | Gln |
| 7 | L-Glutamic acid | Glu |
| 8 | Hydroxytyramine hydrochloride | DA |
| 9 | L-histidine | His |
| 10 | Tryptamine | TrpA |
| 11 | Noradrenaline hydrochloride | NE |
| 12 | Serotonin hydrochloride | 5-HT |
| 13 | L-Tyrosine | Tyr |
| 14 | Adrenaline hydrochloride | E |
| 15 | Kynurenic acid | KynA |
| 16 | 5-Hydroxyindole-3-acetic acid | 5-HIAA |
| 17 | Levodopa | DOPA |
| 18 | L-Tryptophan | Trp |
| 19 | Xanthurenic acid | XA |
| 20 | DL-Kynurenine | Kyn |
| 21 | Vanillymandelic Acid | VMA |
| 22 | 5-Hydroxytryptophan | 5-HTP |
| 23 | Melatonin | MT |
