# Supplementary material for: The DNA methylation status of the serotonin metabolic pathway associated with reproductive inactivation induced by long-light exposure in Magang geese
Source: BMC Genomics. 2023 Jun 26;24:355. doi: 10.1186/s12864-023-09342-0 (PMC10294383; doi:10.1186/s12864-023-09342-0)
Supplement: Supplementary file 4 — Supplementary Material 4 [file 12864_2023_9342_MOESM4_ESM.docx]

| Stage | sample ID | conversion rate (%) | # of unique mapped reads | mapping efficiency （%） | Coverage of C | coverage of C site | | |
| --- | --- | --- | --- | --- | --- | --- | --- | --- |
|  |  |  |  |  |  | CpG | CHG | CHH |
|  | RA-1 | 99.42 | 118,381,158 | 66.9 | 87.3% | 83.85 | 0.78 | 1.00 |
| RA | RA-2 | 99.39 | 113,204,077 | 66.4 | 86.9% | 83.95 | 0.84 | 1.08 |
|  | RA-3 | 99.38 | 108,925,298 | 67.6 | 76.3% | 87.61 | 0.89 | 1.20 |
|  | RD-1 | 99.36 | 123,437,854 | 69.6 | 86.5% | 83.56 | 0.78 | 1.05 |
| RD | RD-2 | 99.39 | 119,649,419 | 68.9 | 86.4% | 83.94 | 0.81 | 1.12 |
|  | RD-3 | 99.39 | 126,677,915 | 71.6 | 88.0% | 83.76 | 0.81 | 1.07 |
|  | RI-1 | 99.38 | 119,020,255 | 66.9 | 87.0% | 83.73 | 0.88 | 1.18 |
| RI | RI-2 | 99.37 | 115,766,275 | 66.6 | 86.5% | 83.91 | 0.72 | 0.98 |
|  | RI-3 | 99.36 | 111,787,701 | 66.5 | 85.9% | 83.75 | 0.76 | 1.05 |

**Table S3 Summary of whole genome bisulfite sequencing data**
