# Supplementary material for: The DNA methylation status of the serotonin metabolic pathway associated with reproductive inactivation induced by long-light exposure in Magang geese
Source: BMC Genomics. 2023 Jun 26;24:355. doi: 10.1186/s12864-023-09342-0 (PMC10294383; doi:10.1186/s12864-023-09342-0)
Supplement: Supplementary file 6 — Supplementary Material 6 [file 12864_2023_9342_MOESM6_ESM.docx]

| Stage | sample ID | Total reads | # of unique mapped reads | unique mapped rate | Total Mapping rate |  |
| --- | --- | --- | --- | --- | --- | --- |
|  |  |  |  |  |  |  |
|  | RA-1 | 27,165,564 | 24,409,110 | 89.85% | 95.99% |  |
| RA | RA-2 | 24,022,219 | 21,425,344 | 89.19% | 95.54% |  |
|  | RA-3 | 22,986,684 | 20,464,010 | 89.03% | 95.26% |  |
|  | RD-1 | 25,550,373 | 22,770,097 | 89.12% | 95.90% |  |
| RD | RD-2 | 28,778,568 | 25,798,930 | 89.65% | 95.85% |  |
|  | RD-3 | 25,814,665 | 22,939,163 | 88.86% | 95.90% |  |
|  | RI-1 | 26,564,650 | 23,645,211 | 89.01% | 95.55% |  |
| RI | RI-2 | 29,715,590 | 25,881,806 | 87.10% | 94.75% |  |
|  | RI-3 | 25,054,675 | 22,062,657 | 88.06% | 95.43% |  |

**Table S5 Summary of transcriptome sequencing data**
